# Implementation of Patient-Reported Outcomes in a Medical Oncology Setting (the iPROMOS Study): Type II Hybrid Implementation Study

**DOI:** 10.2196/55841

**Published:** 2024-08-27

**Authors:** Natasha Anne Roberts, Anita Pelecanos, Kimberly Alexander, David Wyld, Monika Janda

**Affiliations:** 1 The University of Queensland Centre for Clinical Research Herston Australia; 2 Surgical Treatment and Rehabilitation Service Metro North Health and University of Queensland Herston Australia; 3 QIMR Berghofer Medical Research Institute Herston Australia; 4 School of Nursing Queensland University of Technology Kelvin Grove Australia; 5 Cancer Care Services Royal Brisbane and Women's Hospital Herston Australia; 6 The University of Queensland Clinical School St Lucia Australia

**Keywords:** implementation science, iPARIHS, clinical practice, intervention, implementation, facilitator, facilitation, patient-reported outcomes, patient-reported outcome measures, oncology, symptom, symptoms, detection, investigate, service, services, clinic, clinics, Australia, binary logistic models, regression model, regression models, patient, patients, supportive care

## Abstract

**Background:**

Clinical trials have demonstrated that patient-reported outcome measures (PROMs) can improve mortality and morbidity outcomes when used in clinical practice.

**Objective:**

This study aimed to prospectively investigate the implementation of PROMs in routine oncology. Outcomes measured included improved symptom detection, clinical response to symptom information, and health service outcomes.

**Methods:**

Two of 12 eligible clinics were randomized to implement symptom PROMs in a medical oncology outpatient department in Australia. Randomization was carried out at the clinic level. Patients in control clinics continued with usual care; those in intervention clinics completed a symptom PROM at presentation. This was a pilot study investigating symptom detection, using binary logistic models, and clinical response to PROMs investigated using multiple regression models.

**Results:**

A total of 461 patient encounters were included, consisting of 242 encounters in the control and 222 in the intervention condition. Patients in these clinics most commonly had head and neck, lung, prostate, breast, or colorectal cancer and were seen in the clinic for surveillance and oral or systemic treatments for curative, metastatic, or palliative cancer care pathways. Compared with control encounters, the proportion of symptoms detected increased in intervention encounters (odds ratio 1.05, 95% CI 0.99-1.11; *P*=.08). The odds of receiving supportive care, demonstrated by nonroutine allied health review, increased in the intervention compared with control encounters (odds ratio 3.54, 95% CI 1.26-9.90; *P*=.02).

**Conclusions:**

Implementation of PROMs in routine care did not significantly improve symptom detection but increased the likelihood of nonroutine allied health reviews for supportive care. Larger studies are needed to investigate health service outcomes.

**Trial Registration:**

Australian New Zealand Clinical Trials Registry ACTRN12618000398202; https://tinyurl.com/3cxbemy4

## Introduction

The use of symptom patient-reported outcome measures (PROMs) in stringent randomized clinical trials has been shown to improve oncology outpatient care outcomes. Randomized clinical trials reported on individual patient outcomes such as improved physical functioning, better treatment adherence, self-efficacy, quality of life, and overall survival [1-4]. For health services, the use of PROMs for symptom measurement has led to a reduction in emergency presentations and hospital admissions, without increasing clinical workloads [5-7]. These benefits are thought to be due to improved symptom detection by clinicians enabling better supportive care [8-10].

Despite this evidence and an increased uptake of PROMs in routine care, successful implementation in routine care has been inconsistent due to the complex nature of PROMs interventions [4]. More than 2 decades ago, the review by Greenhalgh and Meadows [11] identified that PROMs are challenging to implement, and this was a persisting concern. Recently, guidelines from the European Society for Medical Oncology identified that evidence informing implementation of PROMs in clinical practice is still scarce and further studies that shed light on the barriers to implementation are needed [4].

In 2017, a prospective pilot study (iPROMOS) was designed to investigate the feasibility of investigating implementation of PROMs in medical oncology outpatient care using a type II hybrid implementation clinical trial method [12]. This study structured its implementation approach around the recommendations of the integrated-Promoting Action on Research Implementation in Health Services (iPARIHS) implementation science framework and found that despite some challenges, implementing PROMs in routine oncology clinics was feasible and acceptable [13]. It also showed that it is important to measure underlying processes to ensure that the intervention was delivered in the way it is intended [14]. It was proposed that since PROMs were successfully implemented, clinical benefits such as improved symptom detection and corresponding supportive care would occur. This paper reports on these clinical outcome results of the iPROMOS study.

## Methods

### Study Design

A hybrid feasibility implementation clinical trial was designed to measure both intervention and implementation outcomes [15]. The trial randomized at the level of the outpatient clinic with preimplementation data compared with that of postimplementation data across 2 clinics, with weeks and clinic as fixed effects [12]. The introduction of the intervention was phased for practical reasons, mainly to limit the demand on local resources. The purpose of this study design was to investigate implementation of PROMs taking into consideration the learnings of previous research identifying randomization at the level of the patient was not optimal [5,11,16]. Implementation design features were structured following the iPARIHS framework [17].

The intervention (defined as the “innovation construct” according to the iPARIHS framework) contained 3 components:

1. Completion of the PROM (the Patient-Reported Outcomes version of the Common Terminology Criteria for Adverse Events [PRO-CTCAE] [18] core set [19]) by patients via a touch screen computer when visiting the outpatient department. The PRO-CTCAE was provided for patient completion using REDCap (Research Electronic Data Capture) electronic survey and data capture web application hosted at the Queensland University of Technology [20,21].

The PRO-CTCAE core set evaluates symptoms of fatigue, insomnia, pain, anorexia, dyspnea, anxiety, depression, peripheral sensory neuropathy, constipation, diarrhea, nausea, and vomiting, generating a summary symptom report of all responses by the patient from that encounter.

1. A generated summary of the patient PROM information was made available to medical, nursing, and other clinical teams who reviewed the patient as a part of the patient encounter. Making the report available was integrated into existing workflows.
2. The response by clinical teams to the generated summary of PROM information was documented on a case report form and the patient’s medical record.

The facilitator supported the implementation of the intervention, including being available in-person to support patients to complete the PROM, and staff to obtain the PROM report, if needed. Facilitation actions have been reported as a part of the study findings [13].

This study took place in the medical oncology outpatient department in a large tertiary teaching and training hospital in Southeast Queensland, within the largest public health service in Australia. The hospital serves as a quaternary referral center with specialist medical oncology, hematology, radiation oncology, surgical oncology specialties, medical imaging, nuclear medicine, pathology services, and a high-acuity clinical research unit.

All patients attending the included clinics were eligible to participate if they provided informed consent. At this hospital, 12 medical oncology clinics were potentially eligible, of which 2 clinics were randomly chosen for this study, using a random draw of clinics names in blank envelopes, first by day of the week (so that the study took place on the same day) and then the clinic type. During the preintervention data collection phase both clinics continued to provide usual care (staff noted symptoms or adverse events for their patients that were discovered during anamnesis in the medical chart) [22]. For each patient encounter, staff members documented patient symptoms and symptom details, and whether a patient had a consultation with allied health in the patient chart, a case report was offered initially but this did not get used. This study was conducted over 20 calendar weeks. Clinic 1 then started with the intervention at week 5, while clinic 2 continued with usual care for another 3 months until week 17 and then started the same intervention [14].

The primary outcome was the proportion of doctors and nursing staff documenting symptoms during each patient encounter. A sample size calculation used estimated effect sizes as published by Berry et al [23]. Given a baseline symptom detection level of 75%, it was estimated that 500 participant encounters would be needed to show improvement by 10% or more with 80% power. This was a feasibility study, and it was not to focus on a difference between a particular outcome and having it powered based on this end point.

Secondary outcomes were the proportion of patient encounters with a response to PROMs information, including whether they received additional supportive care by allied health staff (recorded as seen by allied health); proportion of patient encounters that proceeded to an emergency department presentation; and subsequent hospital admissions.

Exploratory outcomes were added to the analysis. These included (1) an analysis of whether the clinician type (doctor or nurse) had an influence on the clinicians’ response to symptom information, and (2) an analysis of any emergent care, other than an emergency presentation. A sensitivity analysis was performed to assess whether public holidays that fell at calendar weeks 8, 13, and 16 had any effect on findings.

Clinic characteristics and outcomes were summarized using descriptive statistics, including frequency and percentages. To assess whether the intervention resulted in a change to the proportion of symptoms assessed by clinicians and additional supportive care consultations, binary logistic regression models were used. Three separate univariable models were fitted for intervention, week (as continuous measure) and clinic, and all 3 clinician types were included in a multivariable model. *P* values less than .05 were indicated as statistically significant. A fixed effect for clinic was chosen for better statistical efficiency.

Emergent care (patient-initiated ad hoc phone calls to clinics, cancer care coordinators, or additional treatments through outpatients), emergency presentations, and hospital admissions were presented as counts and proportions. The study protocol has been published [12].

### Ethical Considerations

The iPROMOS study protocol underwent full review by the Royal Brisbane & Women’s Hospital Human Research Ethics Committee (HREC/17/QRBW/416). Participants completed PROMs after providing informed consent via the touch screen computer. Participation was voluntary. All participant data were made nonidentifiable and stored on password-protected hospital servers.

## Results

**Figure 1:**
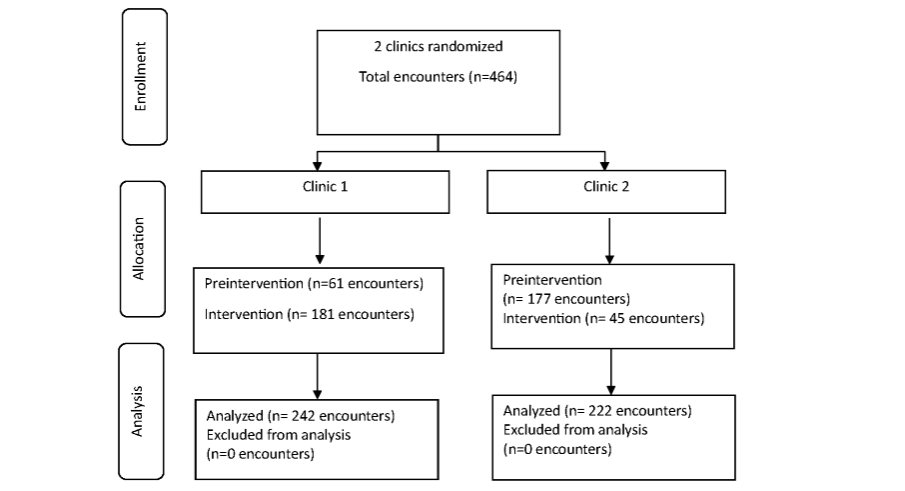
CONSORT (Consolidated Standards of Reporting Trials) diagram for the iPROMOS study.

The consort diagram is presented in Figure 1. There were 464 patient encounters from 421 patients recruited between March and June 2018. The majority of patients (421/464, 91%) had only 1 encounter, while 15 patients had more than 1 (2-7) encounter.

Patient characteristics by intervention period and clinic are shown in Table 1. Reasons for clinic attendance included new patient appointments, intravenous treatments (including standard of care and clinical trials), oral treatments (including standard of care and clinical trials), follow-up, or surveillance for either localized or metastatic disease.

**Table 1:** Patient encounter characteristics by intervention phase and clinic.

			Preintervention (n=242)				Intervention (n=222)		
			Clinic 1 (n=61)		Clinic 2 (n=181)		Clinic 1 (n=177)		Clinic 2 (n=45)
**Treatment, n (%)**									
	None	2 (3)		1 (1)		1 (1)		0 (0)	
	New	5 (8)		27 (15)		26 (15)		2 (4)	
	Intravenous	43 (70)		91 (50)		99 (56)		23 (51)	
	Intravenous clinical trial	2 (3)		2 (1)		23 (13)		4 (9)	
	Oral	1 (2)		18 (10)		6 (3)		6 (13)	
	Oral clinical trial	0 (0)		0 (0)		1 (1)		1 (2)	
	Follow-up	7 (11)		34 (19)		16 (9)		8 (18)	
	Surveillance	1 (2)		8 (4)		5 (3)		1 (2)	
**Stage, n (%)**									
	Localized	44 (75)		82 (46)		120 (69)		19 (42)	
	Metastatic	14 (24)		95 (53)		50 (29)		26 (58)	
	Recurrence	1 (2)		1 (1)		5 (3)		0 (0)	
	Palliative	0 (0)		1 (1)		0 (0)		0 (0)	
**Diagnosis, n (%)**									
	Thyroid	4 (7)		2 (1)		18 (10)		0 (0)	
	Lung	8 (13)		1 (1)		41 (23)		2 (4)	
	Colorectal	0 (0)		4 (2)		0 (0)		0 (0)	
	Breast	0 (0)		50 (28)		0 (0)		10 (22)	
	Prostate	0 (0)		68 (38)		0 (0)		22 (49)	
	Gynecologic	0 (0)		14 (8)		0 (0)		5 (11)	
	Head and neck	49 (80)		0 (0)		117 (66)		0 (0)	
	Genitourinary	0 (0)		42 (23)		1 (1)		6 (13)	

For the primary outcome of symptom detection, 125 of 242 (52%) recorded patient encounters with a doctor had a symptom detected during the preintervention period. During the intervention period, 137 of 222 (62%) patient encounters with doctors had a symptom detected (*P*=.08). For encounters with a nurse, 43 of 97 (44%) identified a symptom before the intervention. During the intervention period, 99 of 130 (76%) documented encounters recorded a symptom detected (*P*=.004).

There was an increase of 3.54 (95% CI 1.26-9.90; *P*=.02) fold in the odds of being seen by allied health in the intervention period compared with the preintervention period after accounting for study week and clinic. A sensitivity analysis of considering week as calendar week instead of study week number (Table S1 in Multimedia Appendix 1) yielded similar results.

Exploratory analyses of symptom detection by clinician type (Table 2) identified that 125 of 242 (52%) recorded patient encounters with a doctor had a symptom detected during the preintervention period, compared with 137 of 222 (62%) patient encounters with doctors during the intervention period. For encounters with a nurse, 43 of 97 (44%) identified a symptom during the preintervention period, and 99 of 130 (76%) identified a symptom during the intervention period. Exploration of models including clinician type indicated that when a doctor identified a symptom (but not nurse) the odds that the patients would be seen by allied health increased during the intervention period (odds ratio 2.32, 95% CI 1.19-4.52; *P*=.013) (Table S2 in Multimedia Appendix 1).

Compared to the preintervention period, emergent care through the service line increased, while emergency presentations and hospital admissions decreased during the intervention period. Before the intervention, there were 57/242 (24%) encounters resulting in emergent care, 27/242 (12%) presenting to an emergency department, and 14/242 (1%) unplanned admissions to hospital. During the intervention period, 60/222 (27%) encounters resulted in required emergent care, 8/222 (0.5%) resulted in presentations to an emergency department, and 8/222 (0.5%) unplanned admissions to hospital.

**Table 2:** Summaries and logistic regression models of symptom identification or referral where week is defined as the study week number.

Total, N														Outcome, n (%)											Unadjusted OR^a,b^ (95% CI)				*P* values							Adjusted OR^c^ (95% CI)					*P* values
														No						Yes																					
**Outcome: doctor identified symptom**																																									
	**Phase^d^**																																								
			Preintervention			242							117 (48)						125 (52)					Reference				.029							Reference					.43	
			Intervention			222							85 (38)						137 (62)					1.51 (1.04-2.18)											1.29 (0.68-2.44)						
	Study week number						464																		1.07 (1.03-1.11)				.001							1.05 (0.99-1.11)					.079
	**Clinic^d^**																																								
			1			238							106 (45)						132 (55)					Reference				.66							Reference					.41	
			2			226							96 (42)						130 (58)					1.09 (0.75-1.57)											1.24 (0.74-2.09)						
**Outcome: nurse identified symptom**																																									
	**Phase ^e^**																																								
			Preintervention			97							54 (56)						43 (44)					Reference				<.001							Reference					.91	
			Intervention			130					31 (24)					99 (76)							4.01 (2.27-7.08)											0.95 (0.37-2.41)							
	Study week number						227																	1.13 (1.06-1.20)				<.001							1.14 (1.04-1.25)					.004	
	**Clinic^e^**																																								
			1					153				41 (27)					112 (73)							Reference				<.001							Reference					<.001	
			2					74					44 (59)						30 (41)					0.25 (0.14-0.45)											0.22 (0.10-0.50)						
**Outcome: seen by allied health**																																									
	**Phase^f^**																																								
			Preintervention			242							231 (95)						11 (5)					Reference				<.001							Reference					.016	
			Intervention			222							179 (81)						43 (19)					5.04 (2.53-10.06)											3.54 (1.26-9.90)						
	Study week number									464															0.99 (0.94-1.05)				.77							0.92 (0.84-1.01)					.069
	**Clinic^f^**																																								
			1			238							187 (79)						51 (21)					Reference				<.001							Reference					<.001	
			2			226							223 (99)						3 (1)					0.05 (0.02-0.16)											0.10 (0.03-0.36)						
**Outcome: allied health identified symptom**																																									
	**Phase^g^**																																								
				Preintervention				11					0 (0)						11 (100)					—^h^						—				—					—		
				Intervention				43					8 (19)						35 (81)					—						—				—					—		
		Study week number					54																								—				—					—	
	**Clinic^g^**																																								
			1						51						8 (16)			43 (84)				—				—							—					—			
					2					3				0 (0)						3 (100)	—								—			—					—				

^a^Unadjusted ORs: each predictor runs as a separate model for each outcome.

^b^OR: odd ratio.

^c^Adjusted OR: all 3 predictors included in 1 model for each outcome.

^d^No: n=202; Yes: n=262.

^e^No: n=85; Yes: n=142.

^f^No: n=410; Yes: n=54.

^g^No: n=8; Yes: n=46.

^h^Not applicable.

## Discussion

Our findings identified that the use of symptom PROMs in oncology outpatient care may potentially benefit clinical and health service outcomes, in line with other clinical studies. As a pilot study, we established that it would be feasible to conduct larger studies. Implementation outcomes were previously reported. We did not identify a statistically significant association between the intervention and symptom identification but there was an association between intervention and the clinical response to symptoms, that is, whether patients received a formal supportive care consultation by allied health. Participants in the intervention period had an increased odds of being seen by allied health compared to those in the preintervention period. This was more likely if a doctor identified a symptom but not if a nurse identified the symptom. While the sample was not powered to detect a significant difference [4], it appears that emergency presentations and unplanned hospital admissions reduced during the intervention period compared with the preintervention period. Emergent care by cancer care coordinators increased during the intervention period.

This study was designed to investigate the structured implementation of a PROMs intervention on clinical outcomes. Larger data sets from clinical practice cohort studies have proven effective in identifying health service benefits of PROMs intervention [6,24-26]. However, the mechanisms underlying these outcomes have not been clear. The use of an implementation science framework, iPARIHS [17], guided implementation and potentially brought to light the clinical mechanisms underlying outcomes. In addition, the use of randomization at the level of clinic aimed to mitigate selection or performance bias, which has been an identified concern in other study designs [5,11]. We identified that more allied health teams and cancer care coordinators were engaged through increased supportive care provision, offering a potential explanation for why the use of PROMs has impacts on health service outcomes.

In this study, symptom detection was not significantly different between the preintervention and intervention groups after the sensitivity analysis, even though the literature has broadly understood that PROMs improve symptom identification [8-10]. This result may be because medical assessment using CTCAE criteria [22] is routine practice for assessing adverse events in the unit and a requirement across the large number of clinical trials recruiting on an ongoing basis. What is also a unique aspect of the setting is cancer care coordinator provision of emergent care in line with an emergency avoidance model of care, where patients will be brought into the outpatients’ department to avoid emergency presentations [27,28]. This service may have also contributed to allied health encounters and health service outcomes and brings attention to the multidisciplinary nature of cancer care.

The electronic system used for patient reporting and clinician review was basic in nature, but it also gave flexibility to tailor it to workflows, communication, and referral processes. The introduction of any technology into a clinical setting can be challenging. For PROMs, this literature supports the use of a simple design with ongoing quality improvement methods to ensure the intervention fidelity [29], which supports the approach taken in this study.

This was a small clinical trial, but it offers learnings for the future. The iPROMOS study highlights the need to understand how to build from a small intense facilitated implementation for scale-up and sustainability. Economic analyses of implementation in such a context of a constantly changing complex clinical environment are needed [29-31]. The potential clinical benefits amplified by the COVID-19 pandemic have forced us to reconsider how clinical care is delivered but ongoing research is needed. The heterogeneity of the populations included in this study indicates that the results may be generalizable [5,32].

The study design was explanatory and documented a series of public holidays impacting the preplanned carrying out of the study across sequential weeks which is a potential limitation in this work. However, a sensitivity analysis did not identify this to affect the results. It cannot be assumed that the presence of symptoms across the phases of this study was equal. Future research could include a reference assessment by a researcher blinded to the intervention. The use of PROMs was not clearly consistently associated with symptom detection across clinician groups. Understanding this variation across clinician groups warrants further research. In addition, patients with head and neck cancer were a larger proportion of one of the randomized clinics. At the facility, these patients are engaged in a multidisciplinary clinic prior to starting treatment and additional funded access to supportive care during treatment. It is unclear whether this contextual factor influenced outcomes. With the large number of patients seen at the facility, and the small number of patients reported in this study, it is possible that the health service outcomes described are random in nature.

## Appendix

Multimedia Appendix 1 Summaries and logistic regression models of symptom identification or referral where week is defined as the calendar week: sensitivity analysis Summaries and logistic regression models of symptom identification or referral using study week.

## Abbreviations

iPARIHS: integrated-Promoting Action on Research Implementation in Health Services
PRO-CTCAE: Patient-Reported Outcomes version of the Common Terminology Criteria for Adverse Events
PROM: patient-reported outcome measure
REDCap: Research Electronic Data Capture

## References

[1] Basch E, Deal AM, Kris MG, Scher HI, Hudis CA, Sabbatini P, Rogak L, Bennett AV, Dueck AC, Atkinson TM, Chou JF, Dulko D, Sit L, Barz A, Novotny P, Fruscione M, Sloan JA, Schrag D (2016). Symptom monitoring with patient-reported outcomes during routine cancer treatment: a randomized controlled trial. J Clin Oncol.

[2] Denis F, Lethrosne C, Pourel N, Molinier O, Pointreau Y, Domont J, Bourgeois H, Senellart H, Trémolières P, Lizée T, Bennouna J, Urban T, El Khouri C, Charron A, Septans AL, Balavoine M, Landry S, Solal-Céligny P, Letellier C (2017). Randomized trial comparing a web-mediated follow-up with routine surveillance in lung cancer patients. J Natl Cancer Inst.

[3] Warrington L, Absolom K, Conner M, Kellar I, Clayton B, Ayres M, Velikova G (2019). Electronic systems for patients to report and manage side effects of cancer treatment: systematic review. J Med Internet Res.

[4] Di Maio M, Basch E, Denis F, Fallowfield L, Ganz P, Howell D, Kowalski C, Perrone F, Stover A, Sundaresan P, Warrington L, Zhang L, Apostolidis K, Freeman-Daily J, Ripamonti C, Santini D, ESMO Guidelines Committee (2022). The role of patient-reported outcome measures in the continuum of cancer clinical care: ESMO clinical practice guideline. Ann Oncol.

[5] Absolom K, Warrington L, Hudson E, Hewison J, Morris C, Holch P, Carter R, Gibson A, Holmes M, Clayton B, Rogers Z, McParland L, Conner M, Glidewell L, Woroncow B, Dawkins B, Dickinson S, Hulme C, Brown J, Velikova G (2021). Phase III randomized controlled trial of eRAPID: eHealth intervention during chemotherapy. J Clin Oncol.

[6] Fromme EK, Eilers KM, Mori M, Hsieh Y, Beer TM (2004). How accurate is clinician reporting of chemotherapy adverse effects? A comparison with patient-reported symptoms from the quality-of-life questionnaire C30. J Clin Oncol.

[7] Billingy N, Tromp V, Aaronson N, Hoek RJA, Bogaard HJ, Onwuteaka-Philipsen BD, van de Poll-Franse L, Hugtenburg JG, Belderbos J, Becker-Commissaris A, van den Hurk CJG, Walraven I, SYMPRO-Lung Consortium (2023). Quality of life after patient-initiated vs physician-initiated response to symptom monitoring: the SYMPRO-Lung trial. J Natl Cancer Inst.

[8] Di Maio M, Gallo C, Leighl NB, Piccirillo MC, Daniele G, Nuzzo F, Gridelli C, Gebbia V, Ciardiello F, De Placido S, Ceribelli A, Favaretto AG, de Matteis A, Feld R, Butts C, Bryce J, Signoriello S, Morabito A, Rocco G, Perrone F (2015). Symptomatic toxicities experienced during anticancer treatment: agreement between patient and physician reporting in three randomized trials. J Clin Oncol.

[9] Laugsand EA, Sprangers MA, Bjordal K, Skorpen F, Kaasa S, Klepstad P (2010). Health care providers underestimate symptom intensities of cancer patients: a multicenter European study. Health Qual Life Outcomes.

[10] Atkinson TM, Li Y, Coffey CW, Sit L, Shaw M, Lavene D, Bennett AV, Fruscione M, Rogak L, Hay J, Gönen Mithat, Schrag D, Basch E (2012). Reliability of adverse symptom event reporting by clinicians. Qual Life Res.

[11] Greenhalgh J, Meadows K (1999). The effectiveness of the use of patient-based measures of health in routine practice in improving the process and outcomes of patient care: a literature review. J Eval Clin Pract.

[12] Roberts NA, Mudge A, Alexander K, Wyld D, Janda M (2019). The iPROMOS protocol: a stepped-wedge study to implement routine patient-reported outcomes in a medical oncology outpatient setting. BMJ Open.

[13] Roberts NA, Janda M, Stover AM, Alexander KE, Wyld D, Mudge A, ISOQOL PROMs/PREMs in Clinical Practice Implementation Science Work Group (2021). The utility of the implementation science framework "Integrated Promoting Action on Research Implementation in Health Services" (i-PARIHS) and the facilitator role for introducing patient-reported outcome measures (PROMs) in a medical oncology outpatient department. Qual Life Res.

[14] Roberts NA, Alexander K, Wyld D, Janda M (2020). Statistical process control assessed implementation fidelity of patient-reported outcome measures (PROMs) in routine care. J Clin Epidemiol.

[15] Pinnock H, Barwick M, Carpenter CR, Eldridge S, Grandes G, Griffiths CJ, Rycroft-Malone J, Meissner P, Murray E, Patel A, Sheikh A, Taylor SJC, StaRI Group (2017). Standards for Reporting Implementation Studies (StaRI) Statement. BMJ.

[16] Basch E (2017). Patient-reported outcomes—harnessing patients' voices to improve clinical care. N Engl J Med.

[17] Harvey G, Kitson A (2016). PARIHS revisited: from heuristic to integrated framework for the successful implementation of knowledge into practice. Implement Sci.

[18] Reeve BB, Mitchell SA, Dueck AC, Basch E, Cella D, Reilly CM, Minasian LM, Denicoff AM, O'Mara AM, Fisch MJ, Chauhan C, Aaronson NK, Coens C, Bruner DW (2014). Recommended patient-reported core set of symptoms to measure in adult cancer treatment trials. J Natl Cancer Inst.

[19] Basch E, Reeve BB, Mitchell SA, Clauser SB, Minasian LM, Dueck AC, Mendoza TR, Hay J, Atkinson TM, Abernethy AP, Bruner DW, Cleeland CS, Sloan JA, Chilukuri R, Baumgartner P, Denicoff A, St Germain D, O'Mara AM, Chen A, Kelaghan J, Bennett AV, Sit L, Rogak L, Barz A, Paul DB, Schrag D (2014). Development of the National Cancer Institute's patient-reported outcomes version of the common terminology criteria for adverse events (PRO-CTCAE). J Natl Cancer Inst.

[20] Harris PA, Taylor R, Thielke R, Payne J, Gonzalez N, Conde JG (2009). Research electronic data capture (REDCap)—a metadata-driven methodology and workflow process for providing translational research informatics support. J Biomed Inform.

[21] Harris PA, Taylor R, Minor BL, Elliott V, Fernandez M, O'Neal L, McLeod L, Delacqua G, Delacqua F, Kirby J, Duda SN, REDCap Consortium (2019). The REDCap consortium: building an international community of software platform partners. J Biomed Inform.

[22] Trotti A, Colevas AD, Setser A, Basch E (2007). Patient-reported outcomes and the evolution of adverse event reporting in oncology. J Clin Oncol.

[23] Berry DL, Hong F, Halpenny B, Partridge AH, Fann JR, Wolpin S, Lober WB, Bush NE, Parvathaneni U, Back AL, Amtmann D, Ford R (2014). Electronic self-report assessment for cancer and self-care support: results of a multicenter randomized trial. J Clin Oncol.

[24] Girgis A, Durcinoska I, Arnold A, Descallar J, Kaadan N, Koh E, Miller A, Ng W, Carolan M, Della-Fiorentina SA, Avery S, Delaney GP (2020). Web-based Patient-Reported Outcome Measures for Personalized Treatment and Care (PROMPT-Care): multicenter pragmatic nonrandomized trial. J Med Internet Res.

[25] Flores LT, Bennett AV, Law EB, Hajj C, Griffith MP, Goodman KA (2012). Patient-reported outcomes vs. clinician symptom reporting during chemoradiation for rectal cancer. Gastrointest Cancer Res.

[26] Greenhalgh J, Gooding K, Gibbons E, Dalkin S, Wright J, Valderas J, Black N (2018). How do patient reported outcome measures (PROMs) support clinician-patient communication and patient care? A realist synthesis. J Patient Rep Outcomes.

[27] Button E, Northfield S, Smith M, Wyld D, Nasato G, Yates P (2020). How do cancer care services in Australia take care of their patients when they require emergency care? We need more evidence. Aust Health Rev.

[28] Northfield S, Button E, Wyld D, Gavin NC, Nasato G, Yates P (2019). Taking care of our own: a narrative review of cancer care services-led models of care providing emergent care to patients with cancer. Eur J Oncol Nurs.

[29] Basch E, Rocque G, Mody G, Mullangi S, Patt D (2023). Tenets for implementing electronic patient-reported outcomes for remote symptom monitoring during cancer treatment. JCO Clin Cancer Inform.

[30] Boulanger M, Petrillo L, Temel J (2023). Listen to the patient: integrating patient-reported outcomes into clinical care. J Natl Cancer Inst.

[31] Roberts NA, Young AM, Duff J (2023). Using implementation science in nursing research. Semin Oncol Nurs.

[32] Schliep ME, Alonzo CN, Morris MA (2018). Beyond RCTs: innovations in research design and methods to advance implementation science. EBP Adv Corner.

