# Supplementary material for: Implementation of Patient-Reported Outcomes in a Medical Oncology Setting (the iPROMOS Study): Type II Hybrid Implementation Study
Source: J Med Internet Res. 2024 Aug 27;26:e55841. doi: 10.2196/55841 (PMC11387919; doi:10.2196/55841)
Supplement: Multimedia Appendix 1 [file jmir_v26i1e55841_app1.docx]

**Table S1. Summaries and logistic regression models of symptom identification or referral where week is defined as the calendar week: sensitivity analysis**

|  | **Total N** | **Outcome No. (%)** | | **Unadjusted OR (95% CI)** | **P** | **Adjusted OR (95% CI)** | **P** |
| --- | --- | --- | --- | --- | --- | --- | --- |
|  |  | **No** | **Yes** |  |  |  |  |
| *Outcome: Doctor identified symptom* | | | | | | | |
|  |  | N = 202 | N = 262 |  |  |  |  |
| Phase |  |  |  |  |  |  |  |
| Pre-intervention | 242 | 117 (48) | 125 (52) | Ref | 0.029 | Ref | 0.39 |
| Intervention | 222 | 85 (38) | 137 (62) | 1.51 (1.04 – 2.18) |  | 1.32 (0.70 – 2.47) |  |
| Calendar week | 464 |  |  | 1.05 (1.02 – 1.09) | 0.001 | 1.04 (0.99 – 1.09) | 0.088 |
| Clinic |  |  |  |  |  |  |  |
| 1 | 238 | 106 (45) | 132 (55) | Ref | 0.66 | Ref | 0.39 |
| 2 | 226 | 96 (42) | 130 (58) | 1.09 (0.75 – 1.57) |  | 1.25 (0.75 – 2.11) |  |
|  |  |  |  |  |  |  |  |
| *Outcome: Nurse identified symptom* | | | | | | | |
|  |  | N = 85 | N = 142 |  |  |  |  |
| Phase |  |  |  |  |  |  |  |
| Pre-intervention | 97 | 54 (56) | 43 (44) | Ref | <0.001 | Ref | 0.93 |
| Intervention | 130 | 31 (24) | 99 (76) | 4.01 (2.27 – 7.08) |  | 1.04 (0.42 – 2.61) |  |
| Study week number | 227 |  |  | 1.10 (1.05 – 1.16) | <0.001 | 1.11 (1.03 – 1.19) | 0.007 |
| Clinic |  |  |  |  |  |  |  |
| 1 | 153 | 41 (27) | 112 (73) | Ref | <0.001 | Ref | <0.001 |
| 2 | 74 | 44 (59) | 30 (41) | 0.25 (0.14 – 0.45) |  | 0.23 (0.10 – 0.52) |  |
|  |  |  |  |  |  |  |  |
| *Outcome: Seen by allied health* | | | | | | | |
|  |  | N = 410 | N = 54 |  |  |  |  |
| Phase |  |  |  |  |  |  |  |
| Pre-intervention | 242 | 231 (95) | 11 (5) | Ref | <0.001 | Ref | 0.013 |
| Intervention | 222 | 179 (81) | 43 (19) | 5.04 (2.53 – 10.06) |  | 3.50 (1.30 – 9.43) |  |
| Study week number | 464 |  |  | 0.99 (0.94 – 1.04) | 0.61 | 0.93 (0.87 – 1.00) | 0.051 |
| Clinic |  |  |  |  |  |  |  |
| 1 | 238 | 187 (79) | 51 (21) | Ref | <0.001 | Ref | <0.001 |
| 2 | 226 | 223 (99) | 3 (1) | 0.05 (0.02 – 0.16) |  | 0.10 (0.03 – 0.36) |  |
|  | | | | | | | |
| *Outcome: Allied health identified symptom* | | | | | | | |
|  |  | N = 8 | N = 46 |  |  |  |  |
| Phase |  |  |  |  |  |  |  |
| Pre-intervention | 11 | 0 (0) | 11 (100) |  |  |  |  |
| Intervention | 43 | 8 (19) | 35 (81) |  |  |  |  |
| Study week number | 54 |  |  |  |  |  |  |
| Clinic |  |  |  |  |  |  |  |
| 1 | 51 | 8 (16) | 43 (84) |  |  |  |  |
| 2 | 3 | 0 (0) | 3 (100) |  |  |  |  |

**Table S2. Summaries and logistic regression models of symptom identification or referral using study week**

|  | **Total N** | **Outcome No. (%)** | | **Unadjusted OR (95% CI)** | **P** | **Adjusted OR (95% CI)** | **P** |
| --- | --- | --- | --- | --- | --- | --- | --- |
|  |  |  | |  |  |  |  |
|  |  | **No** | **Yes** |  |  |  |  |
| *Outcome: Doctor identified symptom* | | | | | | | |
|  |  | N = 202 | N = 262 |  |  |  |  |
| Phase |  |  |  |  |  |  |  |
| Pre-intervention | 242 | 117 (48) | 125 (52) | Ref | 0.029 | Ref | 0.46 |
| Intervention | 222 | 85 (38) | 137 (62) | 1.51 (1.04 – 2.18) |  | 1.28 (0.67 – 2.42) |  |
| Week | 464 |  |  | 1.07 (1.03 – 1.11) | 0.001 | 1.05 (0.99 – 1.11) | 0.10 |
| Clinic |  |  |  |  |  |  |  |
| 1 | 238 | 106 (45) | 132 (55) | Ref | 0.66 | Ref | 0.50 |
| 2 | 226 | 96 (42) | 130 (58) | 1.09 (0.75 – 1.57) |  | 1.20 (0.71 – 2.02) |  |
| Doctor |  |  |  |  |  |  |  |
| Consultant | 229 | 111 (48) | 118 (52) | Ref | 0.038 | Ref | 0.073 |
| Registrar | 234 | 91 (39) | 143 (61) | 1.48 (1.02 – 2.14) |  | 1.41 (0.97 – 2.05) |  |
|  |  |  |  |  |  |  |  |
| *Outcome: Seen by allied health* | | | | | | | |
|  |  | N = 410 | N = 54 |  |  |  |  |
| Phase |  |  |  |  |  |  |  |
| Pre-intervention | 242 | 231 (95) | 11 (5) | Ref | <0.001 | Ref | 0.018 |
| Intervention | 222 | 179 (81) | 43 (19) | 5.04 (2.53 – 10.06) |  | 3.54 (1.24 – 10.10) |  |
| Week | 464 |  |  | 0.99 (0.94 – 1.05) | 0.77 | 0.90 (0.82 – 0.99) | 0.030 |
| Clinic |  |  |  |  |  |  |  |
| 1 | 238 | 187 (79) | 51 (21) | Ref | <0.001 | Ref | <0.001 |
| 2 | 226 | 223 (99) | 3 (1) | 0.05 (0.02 – 0.16) |  | 0.09 (0.03 – 0.34) |  |
| Doctor identified symptom |  |  |  |  |  |  |  |
| No | 202 | 186 (92) | 16 (8) | Ref | 0.031 | Ref | 0.013 |
| Yes | 262 | 224 (86) | 38 (15) | 1.97 (1.07 – 3.65) |  | 2.32 (1.19 – 4.52) |  |
|  |  |  |  |  |  |  |  |
| *Outcome: Seen by allied health* | | | | | | | |
|  |  | N = 410 | N = 54 |  |  |  |  |
| Phase |  |  |  |  |  |  |  |
| Pre-intervention | 242 | 231 (95) | 11 (5) | Ref | <0.001 | Ref | 0.048 |
| Intervention | 222 | 179 (81) | 43 (19) | 5.04 (2.53 – 10.06) |  | 3.49 (1.01 – 12.05) |  |
| Week | 464 |  |  | 0.99 (0.94 – 1.05) | 0.77 | 0.93 (0.83 – 1.04) | 0.18 |
| Clinic |  |  |  |  |  |  |  |
| 1 | 238 | 187 (79) | 51 (21) | Ref | <0.001 | Ref | 0.039 |
| 2 | 226 | 223 (99) | 3 (1) | 0.05 (0.02 – 0.16) |  | 0.18 (0.04 – 0.92) |  |
| Nurse identified symptom |  |  |  |  |  |  |  |
| No | 85 | 76 (89) | 9 (11) | Ref | 0.045 | Ref | 0.47 |
| Yes | 142 | 112 (79) | 30 (21) | 2.26 (1.02 – 5.03) |  | 1.38 (0.57 – 3.32) |  |
|  | | | | | | | |

Confidence interval (CI), odds ratio (OR)
